# A Systematic Protein Refolding Screen Method using the DGR Approach Reveals that Time and Secondary TSA are Essential Variables

**DOI:** 10.1038/s41598-017-09687-z

**Published:** 2017-08-24

**Authors:** Yuanze Wang, Niels van Oosterwijk, Ameena M. Ali, Alaa Adawy, Atsarina L. Anindya, Alexander S. S. Dömling, Matthew R. Groves

**Affiliations:** University of Groningen, Department of Drug Design, A. Deusinglaan 1, 9713 AV Groningen, The Netherlands

## Abstract

Refolding of proteins derived from inclusion bodies is very promising as it can provide a reliable source of target proteins of high purity. However, inclusion body-based protein production is often limited by the lack of techniques for the detection of correctly refolded protein. Thus, the selection of the refolding conditions is mostly achieved using trial and error approaches and is thus a time-consuming process. In this study, we use the latest developments in the differential scanning fluorimetry guided refolding approach as an analytical method to detect correctly refolded protein. We describe a systematic buffer screen that contains a 96-well primary pH-refolding screen in conjunction with a secondary additive screen. Our research demonstrates that this approach could be applied for determining refolding conditions for several proteins. In addition, it revealed which “helper” molecules, such as arginine and additives are essential. Four different proteins: HA-RBD, MDM2, IL-17A and PD-L1 were used to validate our refolding approach. Our systematic protocol evaluates the impact of the “helper” molecules, the pH, buffer system and time on the protein refolding process in a high-throughput fashion. Finally, we demonstrate that refolding time and a secondary thermal shift assay buffer screen are critical factors for improving refolding efficiency.

## Introduction

The demand for innovative protein-based therapeutics to address drug-resistant diseases is ever more pressing. Although the Human Genome Project completed in 2004 has provided a wealth of biological targets for exploration by structural biology and therapeutic drug design, the number of human protein structures determined by NMR spectroscopy, X-ray crystallography and cryoelectron microscopy has not increased dramatically. One of the main obstacles is the availability of large amounts of the target protein. Although *Escherichia coli* (*E. coli*) is a cheap and readily accessible recombinant expression host, up to 70% of eukaryotic proteins expressed in *E. coli* are insoluble as stated by reports from the Center for Eukaryotic Structure Genomics (CESG)^1–5^. Despite this major attrition and the availability of a large number of well-established expression systems (both eukaryotic and bacterial), *E. coli* is still the most widely used expression platform for protein production, due to advantages such as high growth rate in inexpensive medium, rapid biomass accumulation, ease of scale-up and high productivity^6–8^. In addition, a great number of expression vectors, engineered strains and many cultivation strategies are well characterized for the high-level production of heterologous proteins in this organism. Therefore, the development of a systematic refolding method that can be robustly applied for a wide range of protein candidates to efficiently recover correctly folded and biologically active recombinant proteins from inclusion bodies is highly attractive.

Inclusion bodies are composed of aggregates of unfolded, partially folded and misfolded protein. They are often formed due to a lack of chaperones, failing to reach a correct conformation in the reducing environment of the cytoplasm and potentially undergoing proteolytic degradation^9^. Although the protein found in inclusion bodies cannot be directly used for studies due to lack of biological activity, they provide a highly enriched source of target proteins with high purity. Consequently, several refolding methods have been extensively reported (e.g. dilution, dialysis, chromatography and microfluidic chips^10–14^). However, as many proteins can only be refolded under very specific conditions, the development of systematic screening methods that can screen multiple refolding conditions in parallel is still challenging.

Several fractional factorial refolding kits (QuickFold [AthenaES], FoldIt [Hampton research], iFOLD [Novagen] and QuickFold^TM^ Protein Refolding Kit [Molecular Dimensions Limited]) designed to identify optimal refolding conditions are available commercially. Despite this, refolding efforts are still limited by the lack of analytical assays to monitor multiple refolding experiments in parallel. More often, the refolding process is detected by surrogate assays such as turbidity or absorbance that cannot readily discriminate between properly folded and misfolded proteins. Similarly, SDS-PAGE, size exclusion chromatography (SEC) and reversed-phase HPLC assays can solve this problem but are time-consuming and are not compatible with high-throughput methods^15^. Recently, Biter *et al*. demonstrated that differential scanning fluorimetry (DSF) guided protein refolding (DGR) allows for discrimination between folded, misfolded and unfolded states of refolded protein^16^. However, the pH, Anion, Cation crystallization trial (PACT) screen described is optimized for screening protein crystallization conditions rather than protein refolding. The PACT screen DGR based assay evaluates the effect of pH, anions and cations on a sparse matrix basis and does not address widely used refolding agents, such as chaotropes (urea) or aggregation inhibitors (arginine).

In this study, we have developed a systematic buffer screen that contains a dedicated 96-well primary pH refolding screen, combined with a secondary additive screen to assess other refolding agents (e.g. chaotropes, stabilizing agents, counter-ions and reducing agents) as an extension of the DGR approach. Our research demonstrates that DGR can be applied for determining refolding conditions of proteins that contain more specific and complex buffer compositions. To establish and validate the refolding screen, four different proteins were used: the receptor binding domain of hemagglutinin (HA-RBD), interleukin-17A (IL-17A), mouse double minute 2 homolog (MDM2) and the inhibitory receptor ligand of programmed death receptor 1 (PD-L1). We describe the development of a dedicated and systematic protocol that is able to evaluate the impact of pH, buffer system, time, redox couples as well as a range of additives on the protein refolding process. We also show that a secondary thermal shift assay (TSA) screen performed on the refolded material provides improved dialysis conditions for the removal of refolding agents.

## Methods

### Protein expression

For protein expression experiments, 5 mL of an *E. coli* overnight culture was used to inoculate 1 L Luria–Bertani broth. The cells were grown at 37 °C with shaking (180 rpm), 1 mM isopropyl-β-D-1-thiogalactopyranoside (IPTG) was added when the OD_600_ reached 0.6. After induction, the temperature of the culture was lowered to 18 °C and the cells were incubated overnight. The cells were subsequently harvested by centrifugation at 5,000 g for 20 min.

### Isolation of inclusion bodies from cell pellets

The cell pellets were resuspended in lysis buffer (50 mM Tris-HCl, pH 8.0; 300 mM NaCl; 5% glycerol; 3 mM β-mercaptoethanol (β-ME) and 5 mM ethylenediaminetetraacetic acid (EDTA)). The cell suspension was sonicated for 2 min and 2 mM MgSO_4_, 0.01 mg/mL DNAse and 0.1 mg/mL lysozyme were added. The solution was incubated at room temperature for 15 min. Cell debris was removed by centrifugation (19,000 g). The resuspension, sonication and centrifugation procedure was repeated 4 times using lysis buffer with 0.5% Triton X-100. The inclusion bodies were incubated in lysis buffer with 0.5% Triton X-100 overnight. Finally, the inclusion bodies were washed twice in wash buffer (50 mM Tris-HCl, pH 8.0; 300 mM NaCl; 5% glycerol; 3 mM β-ME and 5 mM EDTA).

### Solubilisation/denaturation of proteins from inclusion bodies

Inclusion bodies were resuspended in either 8 M urea or 6 M guanidine-HCl. Insoluble material was removed by centrifugation at 19,000 g for 20 min.

### Refolding screen in 96-well format

The denatured target protein was refolded by shock dilution at a ratio of 1:20. 10 μL of denatured protein solution at ~5 mg/mL was added into each well of a 96-well plate. Subsequently, 190 μL of refolding buffer from a pre-prepared master plate was transferred to the plate containing the denatured protein. Each well contained a final volume of 200 μL. After set up, the plate was incubated with gentle shaking at room temperature or 4 °C.

### Detection of a refolding buffer by DGR

For the 96-well refolding detection assay, a 25x dilution of the 5000x SYPRO Orange stock (Invitrogen) was prepared in water. For each well, 5 μL of this diluted SYPRO Orange and 45 μL of the refolding sample was added. The plate was covered with sealing tape and centrifuged at 600 g for 1 min to remove any air bubbles. A BioRad CFX96 RT-PCR machine was programmed with a 3 min equilibration time at 22 °C. Subsequently, the plate was heated from 22 to 90 °C with stepwise increments of 0.5 °C per 30 s, followed by the fluorescence reading optimized for SYPRO Orange at 485 nm (Excitation) and 530 nm (Emission) after each step. The 200 μL refolding sample prepared allows for the refolding experiments to be analyzed by DGR at four different time points, aiding the identification of the optimal incubation time for the refolding process.

### Scale-up refolding for Protein stability buffer screen

The subsequent step uses a TSA analysis to screen for dialysis conditions to remove refolding agents. 2.5 mL of denatured protein sample (~12.5 mg) was added dropwise into 50 mL of the refolding buffer identified above, under continuous stirring. The mixture was incubated under stirring for a period of time (as identified from the time points tested in the previous screen). The optimal buffer for subsequent purification and/or concentration could then be identified using a more standard thermal shift assay approach (DSF/TSA)^17^. Briefly, samples from the up-scaled refolding step were concentrated to a maximum of 1 mg/mL and spun to remove any precipitation (5 minutes at 18,000 g). SYPRO Orange (Invitrogen) was prepared by diluting 2.5 μL of the stock solution in DMSO into 500 μL of the concentrated refolded protein sample. For each well in the 96-well plate, 5 μL of protein/SYPRO Orange solution was diluted with 45 μL of the buffer screen formulation. Then the plate was centrifuged at 600 g for 1 min before the start of the assay to remove any air bubbles. The TSA analysis was performed as described above.

### Scale-up refolding for Protein production

After identification of a suitable dialysis buffer, scale-up refolding was repeated and the refolded sample was dialyzed against the dialysis buffer identified. The dialyzed sample was then concentrated to 20 mL using an Amicon 6050 concentrator and further concentrated by a Vivaspin 20 centrifugal concentrator (Sartorius) at 2,000 RCF at 4 °C.

### Size Exclusion Chromatography (SEC) and Static light Scattering (SLS)

The concentrated proteins were purified via SEC using a Superdex 200 10/300 or HiLoad 16/60 Superdex 75 column (GE Healthcare) pre-equilibrated with the identified dialysis buffer using an NGC liquid-chromatography system (Bio-Rad). Where necessary, the molecular masses of the proteins in solution were determined using static light scattering (SLS). A miniDAWN TREOS from Wyatt technology was connected in line with the NGC system. Proteins were concentrated to 2 mg/mL before injection.

## Results

### Development of a Dedicated Refolding Screen

**Figure 1:**
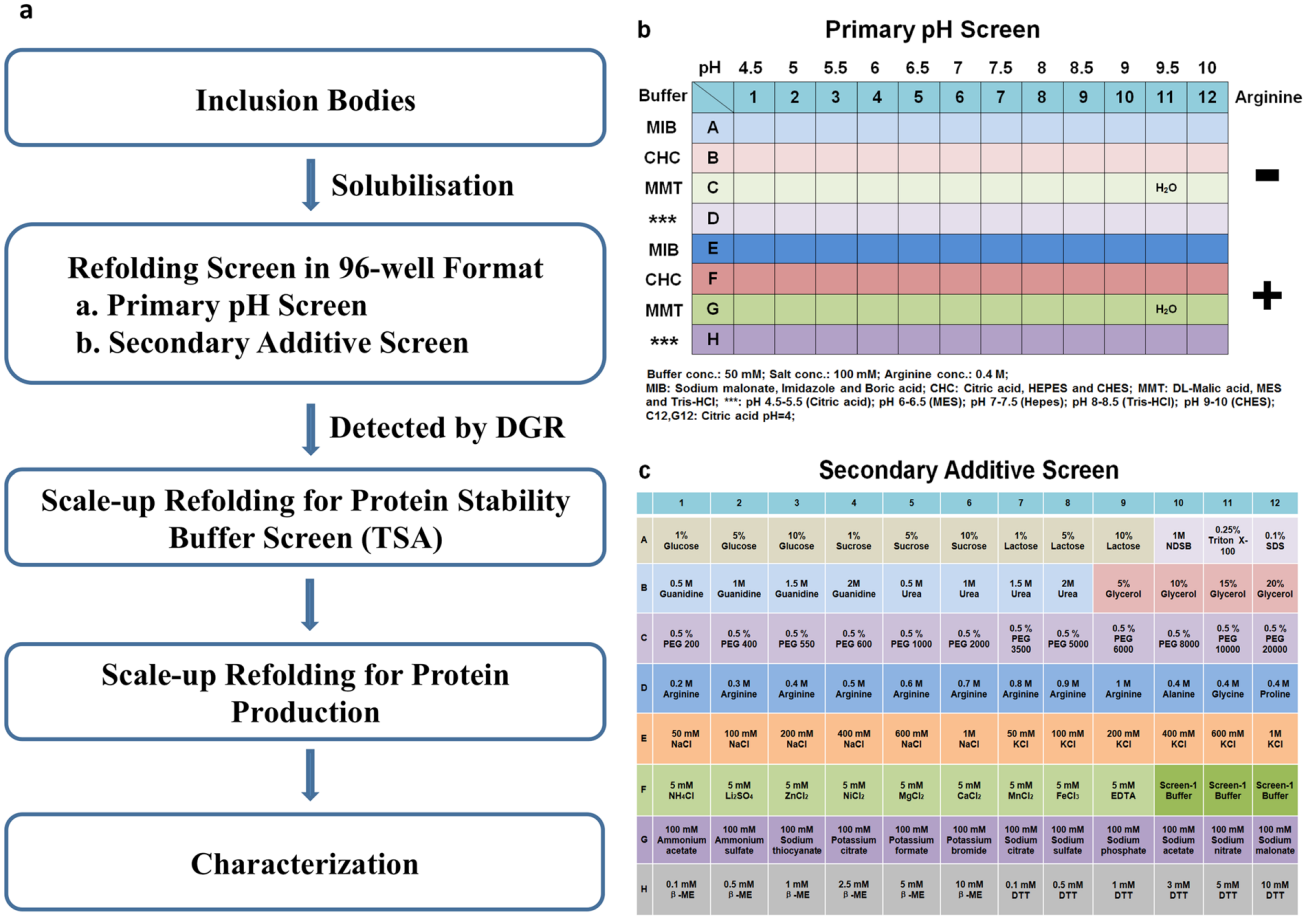
(**a**) Process flow chart: The inclusion bodies of the protein of interest were first purified and solubilized. This was followed by a primary pH refolding screen in 96-well plate, detected the refolding by DGR at different time points. From this screen, initial refolding conditions (pH/Buffer system/Arginine) can be identified. The initial refolding conditions are then combined with an additive screen and assayed using the same procedure to identify the final refolding buffer. Subsequently, protein refolding was scaled up. The first batch of refolded protein was concentrated and directly used for TSA to obtain an optimal protein stability buffer. Finally, the protein refolding was scaled up again and the refolded protein was dialyzed to the protein stability buffer, concentrated and used for characterization. (**b**) Composition of the primary pH screen; Buffer conc.: 50 mM; Salt conc.: 100 mM; Arginine conc.: 0.4 M; MIB: sodium malonate, imidazole and boric acid; CHC: citric acid, HEPES and CHES; MMT: DL-malic acid, MES and Tris-HCl; ***: pH 4.5–5.5 (Citric acid), pH 6–6.5 (MES), pH 7–7.5 (HEPES), pH 8–8.5 (Tris-HCl), pH 9–10 (CHES); C12, G12: Citric acid at pH 4; (**c**) Composition of the secondary additive screen.

Our refolding buffer screen is composed of a “primary pH screen” (Fig. 1b) and a “secondary additive screen” (Fig. 1c). The first screen identifies a “basic buffer” with the optimal pH in the best buffer system for protein refolding; the second screen uses the “basic buffer” in combination with a wide range of additives (mostly protein stabilizers)^18–22^.

### Primary pH Screen

The first part (row A–D) contains a variety of buffer systems in combination with 100 mM NaCl. Similarly, the second part (row E–H) contains these buffers combined with 0.4 M L-arginine hydrochloride, the most commonly used aggregation inhibitor to suppress aggregation and enhance protein refolding^23–29^. This screen covers a pH range from 4.0 to 10.0 (the pH of MMT buffer was 4.0–9.0). Importantly, we use 4 different buffer systems: MIB buffer, CHC buffer, MMT buffer and single buffers. These buffer systems are composed of several reagents, ensuring pH stability over a large range. The different buffer systems not only allow us to explore the effect of pH, but also the impact that different buffer compositions may have on the protein refolding process. The initial DGR screen (Fig. 1b) focuses on the choice of pH and buffer system in the presence or absence of arginine. Information from this screen is used to perform the secondary screen (Fig. 1c), which focuses on identifying further buffer components that will improve refolding efficiency (i.e. chaotropes, stabilizing agents, counter ions and reducing agents).

### Secondary Additive Screen

The optimal condition found in the primary pH screen was used as a standard composition to prepare a secondary additive screen, by diluting the additives into the identified conditions of the primary screen. Conditions A1-A9 are used to analyze the effect of 3 different sugars: glucose, sucrose and lactose at three different concentrations. Conditions A10-A12 contain 3 different detergents, which can bind to protein surfaces to prevent aggregation. Conditions B1-B8 contain 2 different chaotropic agents (0.5–2 M): guanidine-HCl and urea, which are commonly used for dissolving protein inclusion bodies and may promote refolding at lower concentrations. Glycerol and PEG (200–20,000) are commonly used protein stabilizers and are screened in conditions B9-C12. Conditions D1-D12 compare the effect of different concentrations of arginine (0.4–1 M) and 3 amino acids (alanine, glycine and proline). Conditions E1-E12 are included to analyze protein refolding in the presence of NaCl/KCl over a wide concentration range (50–1000 mM). This allows exploration of whether low or high ionic strength will contribute to protein refolding process. Several metal salts with different valences are contained in conditions F1–F9. EDTA was also included in this group to investigate the refolding process in the absence of divalent metals. The buffer found in the primary pH screen in triplicate was contained in F10–F12 to ensure that the results from the assay are robust and reliable. G1–G12 is used to evaluate the effect of 12 different salts. Finally, two different reducing agents (β -ME and Dithiothreitol (DTT)) in several concentrations are included in H1-H12.

### HA-RBD

**Figure 2:**
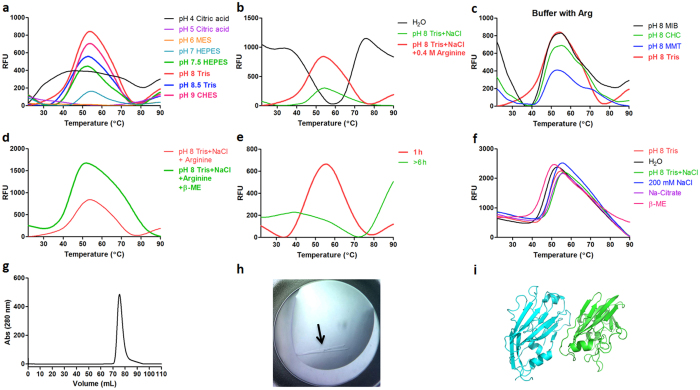
DGR of HA-RBD. (**a**) Melting transition curves of samples in single buffer at different pH values. (**b**) The impact of arginine on refolding. (**c**) Comparison of sample refolded in different buffer systems at pH 8 in the presence of arginine. (**d**) Melting transition curves of samples in the presence of the additive β-ME. (**e**) Melting transition curves collected at different time points. (**f**) Results of TSA buffer screen. (**g**) Size exclusion chromatogram of refolded HA-RBD. (**h**) Crystals of HA-RBD and (**i**) Molecular replacement solution of refolded HA-RBD.

The receptor-binding domain of Hemagglutinin (HA-RBD) is one of the major surface proteins of the influenza virus and plays an important role at the beginning of the viral cycle^30, 31^. HA-RBD is located within residues 63–286 of the influenza virus H1N1 and has a molecular weight of approximately 25 kDa, containing 4 cysteine residues. A synthetic gene corresponding to A/H1N1/2009 influenza virus hemagglutinin was cloned into the pETM11 vector^32^ and expressed in *E. coli* BL21* (DE3) cells. The inclusion bodies were denatured in Tris-HCl buffered 8 M urea solution (pH 8) at a protein concentration of 5 mg/mL. The denatured inclusion bodies were first submitted to the pH refolding screen. The results showed that pH and the presence of arginine play a key role in the refolding process. Firstly, when refolding experiments were performed over a pH range in single buffers (Fig. 1b, Row H), a clear melting transition signal was seen for pH values above 7.5 (Fig. 2a). Further inspection revealed that the addition of arginine to the refolding buffer resulted in a further improvement of the melting curves (Fig. 2b). Finally, it is clear that the single buffer system using Tris-HCl pH 8.0 gave the best refolding signal (Fig. 2c). As the T_M_ values estimated from the curves shown in (Fig. 2a–c) are very similar (~45 °C), refolding efficiency was judged by the highest fluorescence intensity and lowest initial background. Thus, our initial screen identified an optimal pH, buffer component and additive for the refolding of HA-RBD (Tris-HCl pH 8.0 and 0.4 M arginine).

This buffer was then used as a standard addition in follow-up refolding experiments using the secondary additive screen (Fig. 1c). These experiments demonstrated that only the addition of β-ME resulted in an improved signal (Fig. 2d). All other conditions screened by plate 2 resulted in either no effect or a reduction in the refolding process as judged from the melting curves. This includes an analysis of increasing salt concentrations (Fig. 1: Plate 2: Row E), suggesting that 100 mM NaCl represented the optimal ionic strength for refolding.

Our results also showed that refolding time has a substantial impact on the refolding process (Fig. 2e). When the protein was refolded over 1 h, clear melting transition signals could be measured. Surprisingly, when the refolding time was ~6 h, the melting transition signals disappeared. This conclusion is also confirmed by the preparative-scale (50 mL) refolding experiment. Initially, the refolding solution was clear, while after 1 h the refolding solution showed minor precipitation, increasing to larger degrees of precipitation at 6 h.

The above analysis resulted in the identification of a suitable refolding buffer (50 mM Tris-HCl pH 8, 100 mM NaCl, 0.4 M arginine, 3 mM β-ME). A second DSF-based screen (TSA buffer screen), similar in approach to that used in other laboratories^17^, was used to identify an dialysis buffer for the removal of refolding agents. From this screen, four key factors were found to stabilize the refolded protein: 50 mM Tris buffer at pH 8, 200 mM sodium chloride and 10 mM sodium citrate (Fig. 2f). Surprisingly, while the presence of β-ME was shown to be a positive factor in the refolding of HA-RBD, this second analysis demonstrated that the presence of β-ME was a negative factor on HA-RBD stability once it is refolded. When the sample was dialyzed to this new buffer, the protein could be easily concentrated to 7 mg/mL, whereas the protein dialyzed into 50 mM Tris-HCl, pH 8, 100 mM NaCl and 3 mM β-ME (ie. the refolding buffer omitting arginine) could not be concentrated above 0.5 mg/mL. The increase in ionic strength and addition of citrate ions seem to play a very important role in protein stabilization after the removal of arginine.

Refolded HA-RBD appeared homogeneous via size-exclusion chromatography and we were able to crystallize the refolded HA-RBD (100 mM Tris-HCl, pH 8.8 and 36% PEG 2000) in the space group *P*2_1_, with unit-cell parameters *a* = 39.5 Å; *b* = 73.7 Å; *c* = 73.7 Å and α = 90°; β = 100.72°; γ = 90° (Fig. 2g)^33^. The structure was solved to 1.9 Å resolution (Fig. 2h and i).

### IL-17A

**Figure 3:**
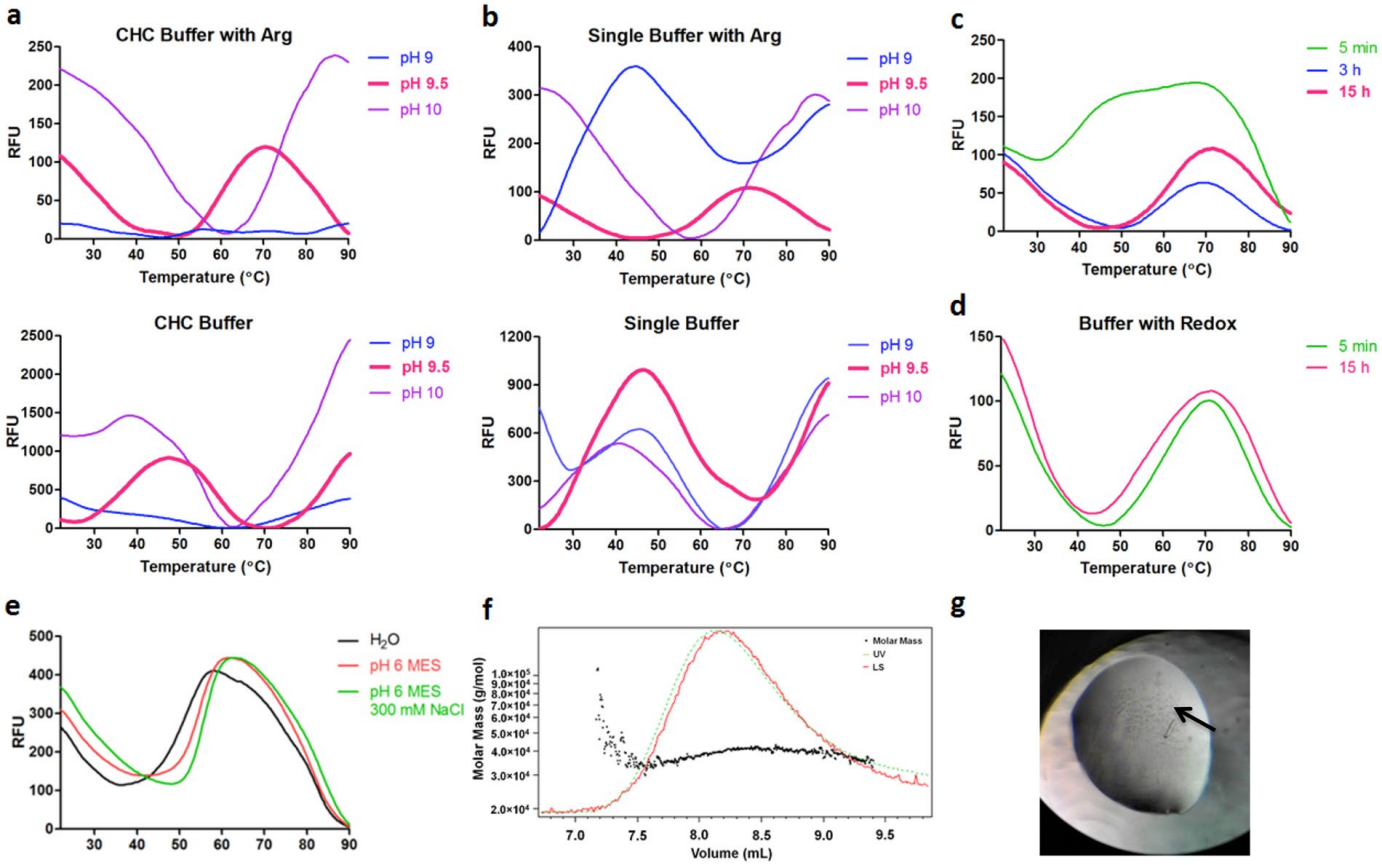
DGR of IL-17A. (**a**) Melting transition curves of IL-17A in CHC buffer at pH 9–10 in the presence or absence of arginine. (**b**) Melting transition curves of IL-17A in single buffer at pH 9–10 in the presence or absence of arginine. (**c**) Melting transition curves collected at different time points in CHES pH 9.5, 0.4 M Arg. (**d**) Melting transition curves in CHES pH 9.5 in the presence of redox agents. (**e**) Results of the TSA buffer screen. (**f**) Characterization of refolded IL-17A by SLS, demonstrating presence of dimer. (**g**) Crystal of IL-17A.

Interleukin-17A (IL-17A), produced by Th17 cells, has been implicated in the pathogenesis of various autoimmune diseases such as rheumatoid arthritis^33^. A synthetic codon optimized gene encoding human IL-17A was also cloned into a pETM11 expression vector and expressed in *E. coli* BL21* (DE3) cells. The inclusion bodies were denatured in a buffer containing 20 mM Tris pH 8.5, 6 M guanidine-HCl, 100 mM NaCl, 5 mM EDTA and 10 mM DTT. From the first pH refolding screen, it is clear that pH and choice of buffer system have a substantial effect on the refolding of IL-17A. Results showed that IL-17A only gave a clear melting transition signal at pH 9.5 (Fig. 3a and b). At this pH, the CHC and CHES buffer systems showed a refolding signal while the MMT and MIB buffer systems did not. By comparing the refolding curve in CHES refolding buffer with and without arginine at pH 9.5, it is clear that arginine was also an indispensable additive for the refolding of IL-17A (Fig. 3b). In the presence of arginine, both refolding curves in the two buffer systems showed a T_M_ at around 60 °C. However, the T_M_ was decreased to around 40 °C when the arginine was removed, suggesting that IL-17A was misfolded at pH 9.5 without arginine.

Refolding time was also found to play an important role in the refolding of IL-17A (Fig. 3c). Three time points (5 min, 3 h and 15 h) were selected to evaluate the impact of time on the refolding of IL-17A. No melting transition signals were found when the protein was refolded for 5 min. However, when the refolding time is over 3 h, a melting transition signal can be detected.

As a result of this analysis, 50 mM CHES at pH 9.5, 100 mM NaCl, 0.4 M arginine were selected as the refolding buffer. A subsequent TSA assay demonstrated that IL-17A was more stable in MES buffer (pH 6) than CHES Buffer (pH 9.5) (Fig. 3e). In addition, increasing the ionic strength (NaCl) from 100 mM to 300 mM also contributes to the stability of IL-17A. While immediate dialysis of IL-17A against MES buffer at pH 6 resulted in a large degree of precipitation, the refolding yield was improved to 50% by step-wise dialysis of the refolded material against buffers at pH 9, pH 8 and finally pH 6, all at 300 mM NaCl.

As IL-17A has been previously refolded in buffer with redox couples, the impact of them on the refolding was further explored^34^. By adding 5 mM cysteine and 0.5 mM cystine to the refolding buffer described above, we were surprised to see a clear transition curve when the protein was incubated in refolding buffer for only five min (Fig. 3d). In addition, the T_M_ and fluorescence intensity of the refolding curve did not further increase when the protein was refolded for 15 h. This suggests that the redox shuffling system significantly increases the refolding rate of IL-17A, while having little impact on the refolding yield.

Recombinant IL-17A was successfully refolded as a homodimer from inclusion bodies as confirmed by SLS (Fig. 3f). The SLS experiment showed that the average molecular mass of refolded protein was 38.6 kDa (±3.515%) with a polydispersity of 1.005 Mw/Mn (±4.997%), in agreement with previously published results (dimeric IL-17A)^35^. The sample was also successfully crystallized in 100 mM Sodium citrate, pH 5.5, 9% 2-propanol and 10% PEG 10000 (Fig. 3g), conditions similar to those reported previously^35^.

### MDM2

**Figure 4:**
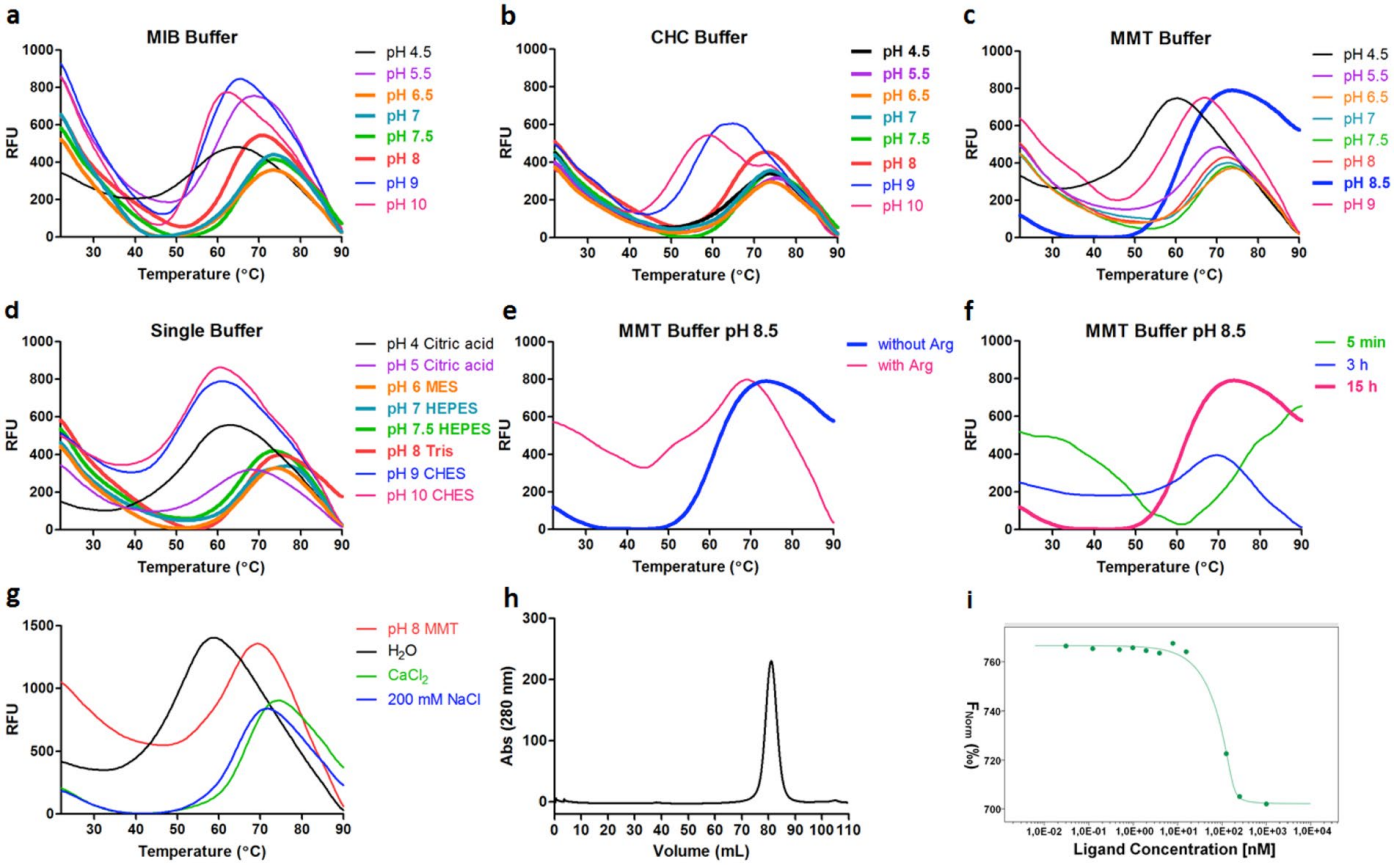
DGR of MDM2. Melting transition curves of MDM2 in (**a**) MIB buffer (**b**) CHC buffer (**c**) MMT buffer and (**d**) single buffer in the absence of arginine. (**e**) The impact of arginine on the refolding. (**f**) Melting transition curves collected at different time points. (**g**) Results of TSA buffer screen. (**h**) Size exclusion chromatogram of refolded MDM2. (**i**) MST binding curve of MDM2 against a known inhibitor.

MDM2, the negative regulator of transcription factor p53, has emerged as a novel non-genotoxic target for acute myeloid leukemia (AML) treatment^36^. A gene encoding MDM2 in pETM 20(b+) expression vector (a kind gift of Prof. Tad A. Holak, Jagiellonian University, Poland) was expressed in *E. coli* BL21* (DE3). The inclusion bodies were dissolved in a buffer containing 20 mM Tris pH 8.5, 6 M Guanidine-HCl, 100 mM NaCl, 5 mM EDTA and 100 mM DTT. The refolding screen was first conducted at room temperature, however, no clear transition curves was shown by DGR. Then the refolding was further explored at 4 °C. In sharp contrast with IL-17A, MDM2 showed transition signals in nearly all conditions of the buffer systems at a lower temperature (Fig. 4a–d). Based on an initial analysis of the DGR curves, refolding was attempted in HEPES at pH 7.5. However, this was unsuccessful as the protein precipitated readily after refolding. A re-analysis of the DGR curves suggested that refolding performed in the MMT buffer at pH 8.5 (Fig. 4c) showed the lowest degree of misfolding, as judged from the initial fluorescence intensity. Unlike HA-RBD and IL-17A, the presence of arginine does not improve the refolding of MDM2. The total fluorescence intensity became lower and the initial fluorescence background increased when the arginine was added (Fig. 4e). Subsequent refolding attempts using arginine as an additive also demonstrated that the protein readily precipitated under these conditions.

As a result, 50 mM MMT buffer and 100 mM NaCl at pH 8.5 was chosen as the refolding buffer and refolding time was 15 h (Fig. 4f). A subsequent TSA assay demonstrated that pH 8 and 200 mM NaCl provided more stability than the refolding buffer for the refolded sample (Fig. 4g). In addition, 1 mM CaCl_2_ was shown to stabilize the protein. As predicted, MDM2 shows improved stability in this buffer (50 mM MMT buffer pH 8 and 200 mM NaCl, 1 mM CaCl_2_). Correct folding of MDM2 is indicated by size-exclusion chromatography and strong binding to a known inhibitor of the MDM2:p53 protein-protein interaction (Fig. 4h and i).

### PD-L1

**Figure 5:**
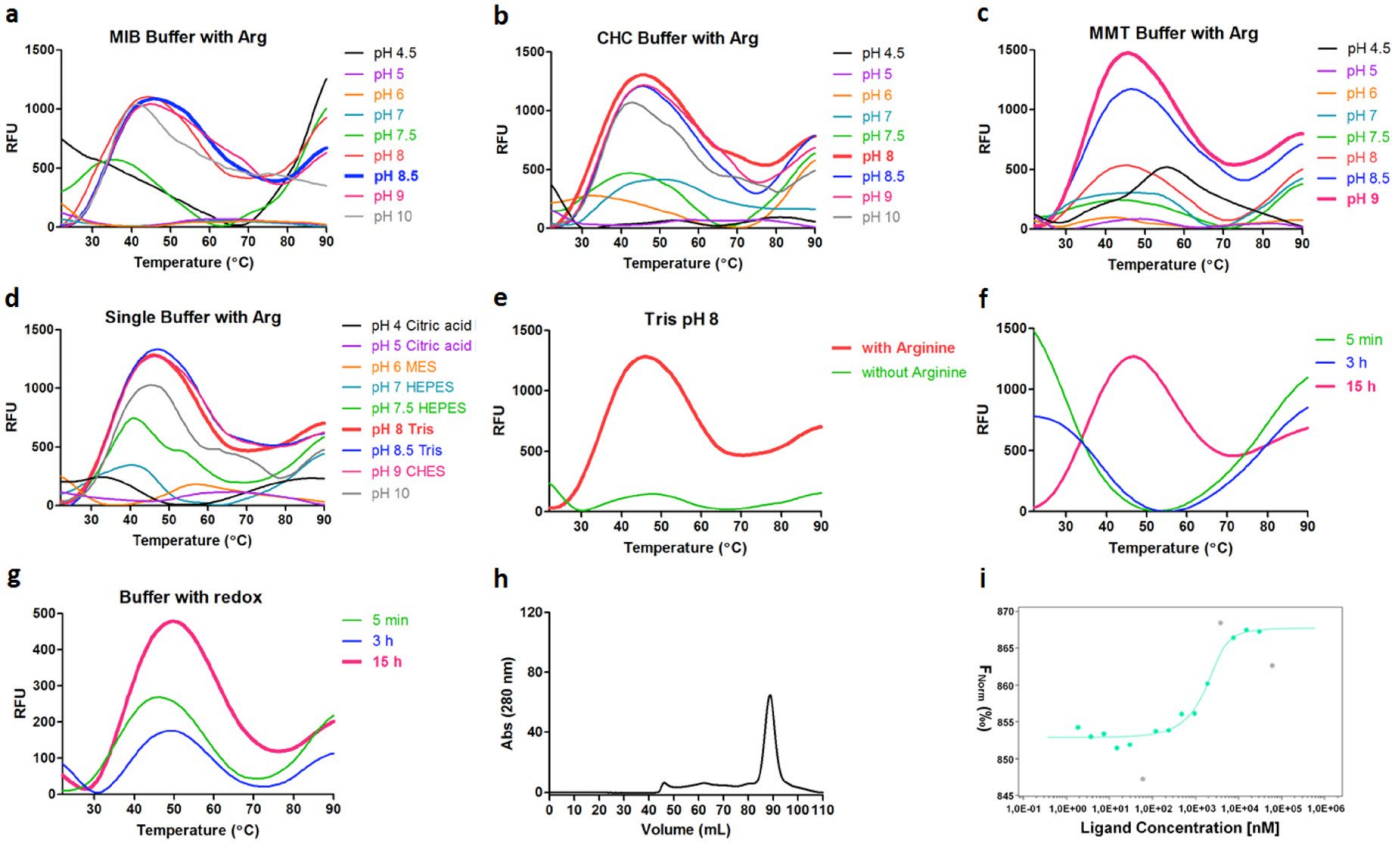
DGR of PD-L1. Melting transition curves of PD-L1 in (**a**) MIB buffer (**b**) CHC buffer (**c**) MMT buffer and (**d**) single buffer at different pH value in the presence of arginine. (**e**) The impact of arginine on the refolding. (**f**) Melting transition curves detected at different time points. (**g**) Melting transition curves detected at different time points in buffer with a redox couple. (**h**) Size exclusion chromatogram of refolded PD-L1. (**i**) MST binding curve of PD-L1 and PD-1.

The inhibitory receptor ligand of programmed death receptor 1 (PD-L1), which plays an important role in the maintenance of peripheral immune tolerance, has become a very promising target for the treatment of melanoma and other types of cancer^37, 38^. A partial gene encoding for the interacting extracellular domains of human PD-L1 (amino acids 18–134) was cloned into a pET21b expression vector (a kind gift of Prof. Tad A. Holak, Jagiellonian University, Poland) and expressed in *E. coli* Rosetta 2(DE3) pLysS. The inclusion bodies were denatured in buffer containing 50 mM MES (pH 8.0), 10 mM Na-EDTA, 1 M urea, 6 M guanidine-HCl and 1 mM DTT. As shown in Fig. 5a,b,c and d, apparent transition signals were observed over a wide range of pHs among the buffer systems in the presence of arginine. In the absence of arginine, only weak transition signals could be detected (Fig. 5e). When the refolding pH is lower than 7.5, a significant reduction in the fluorescence intensity can be seen (Fig. 5d). In an MMT refolding buffer, a clear transition signal is also detected at pH values above 8, and fluorescence intensity increased as the pH increased (Fig. 5c).

The results also showed that the refolding incubation time has an influence on the refolding process. From (Fig. 5f), no transition curves are seen when the refolding time was less than 3 h. However, when the refolding time is over 15 h, a clear melting transition signal was shown. Similarly to IL-17A, the influence of a redox couple (0.25 mM Glutathione (GSH)/0.25 mM Glutathione disulfide (GSSG)) was also explored (Fig. 5g). We observed that a clear melting curve was found when the protein was refolded for 3 h and the fluorescence intensity was further increased when the refolding time was 15 h. In this example, the addition of a redox couple again appears to make a significant difference to the refolding rate. However, we cannot exclude the possibility that redox couples may stabilize the final folded state, rather than drive the refolding process. SEC and MicroScale Thermophoresis (MST) experiments showed that PD-L1 was correctly refolded (Fig. 5h and i). The MST results show that the refolded protein was homogenous and binds with PD-1 with an estimated K_D_ of 0.5 µM.

## Discussion

In the post-genome era, the large proportion of proteins overexpressed in *E. coli* that are sequestered to the insoluble fraction of the cell has become a major bottle neck in the protein structure analysis and therapeutic drug development. Expression in other systems such as yeast, insect cells, mammalian cells and cell-free systems may overcome folding problems and produce biologically active proteins that contain post-translational modifications, but expression in these systems often gives lower yields of the recombinant proteins and the cost is relatively high for large scale protein production. In this context, recovering biologically active proteins from bacterial inclusion bodies is attractive because of the high protein expression yields and low cost. However, there is no universal procedure that satisfies all protein folding requirements to date. In particular, the refolding process is highly limited by the lack of reliable biophysical methods to rapidly detect the formation of correctly refolded protein. In this assay, we established a systematic refolding procedure with the help of the DSF/DGR technique that allows for the detection of the protein refolding in an efficient manner. In addition, the refolding buffer screens we designed allow for the exploration of a wide pH range, different buffer systems, time, temperature as well as a large number of chemical additives in a high throughput manner.

### Refolding pH buffer screen

It is well known that pH plays an important role in protein solubility, underscoring the necessity of exploring a broad pH range for our generalized primary refolding screen. Based on our primary pH screen results, it is clear that we can establish an effective refolding pH for all four proteins investigated. By comparing the DGR signal of our samples, we can also conclude that the effect of the pH on protein refolding is protein-specific. MDM2 showed a very broad refolding pH range, HA-RBD and PD-L1 showed a narrow pH range while IL-17A could only be refolded at a single defined pH.

Although pH is important, it is not the only variable to consider in our primary refolding screen. We also introduced four different buffer systems to the primary screen. The advantage of using different buffer systems was demonstrated by our results. In each case, differences in the type of buffer can be observed. In particular IL-17A could only be successfully refolded following our approach in the CHC or CHES buffers. These results strongly indicate that the reagents composing the buffer system can have an impact on the refolding process and are an essential parameter to screen.

In the presence of arginine, the fluorescence transition signals of HA-RBD and PD-L1 were significantly increased and the T_M_ of IL-17A became significantly higher. Our screen allows the examination of the effect of arginine in combination with different buffer system and is shown to be another essential parameter in successful refolding. Arginine was selected for our screen based on a literature survey that demonstrated that it is the most frequently used “helper” molecule in refolding. While we do not currently explore the use of other frequently used “helper” molecules (such as the detergent Non-Detergents Sulfobetaines (NDSB)), these could be added to the protocol with an expansion of the primary screen and an associated increase in sample/experimental time.

We have also designed a secondary additive screen that consists of different types of protein stabilizers. By screening these additives the refolding yield of HA-RBD could be increased. As the combination of aggregation inhibitor with protein stabilizer has been reported since 1990s, we believe that it is a very promising combinatorial approach to increase refolding yield^39^. Finally, by deconvoluting the effects of pH, ions and additives we can achieve a more efficient screening of the complex chemical space that governs protein refolding.

### Refolding time and redox

Our refolding procedure also allows for detection of the refolding signal at different time points. Our data shows that this can be an essential parameter to monitor. In our study, HA-RBD had the optimal refolding efficiency when the refolding time was lower than 1 h and the protein melting transition signal disappeared after 6 h. IL-17A and MDM2 showed the opposite effect: a weak DSF signal was found at 3 h, while the transition signal increased with time and the highest refolding signal was found at 15 h. A signal for PD-L1 was only observed when the protein was refolded for 15 h. These results indicate that time is also an important factor in refolding efficiency and that initially poor refolding results may not be indicative of the ultimate refolding efficiency in any given buffer.

Our data also confirm that the addition of redox couples can be a critical factor in successful refolding. In two cases (IL-17A and PD-L1) refolding could be detected as soon as 5 minutes incubation in the presence of the “correct” redox couple. However, in both cases successful refolding could be achieved over a longer incubation time in the absence of the redox couples. This indicates that the redox state of the solution may play a role in the rate of refolding of these proteins, but is not necessarily essential. However, we cannot exclude that the redox state is essential for the final stability of the refolded protein.

### Protein stabilization buffer screen

After refolding, it was necessary to remove the “helper molecules”, such as arginine, that might affect the measurement of the protein concentration or other downstream experiments. However, one problem we normally encountered was that the refolded protein is frequently prone to aggregation after the sample was dialyzed to the refolding buffer without the “helper” molecules. This is potentially due to the sample containing a sub-fraction of mis-folded protein or the samples being partially refolded. Indeed it appears from our data that buffers optimal for protein refolding are not necessarily ideal for subsequent sample stability. Hence, we suggest that the simple step of performing a TSA buffer screen to determine an ideal stabilizing buffer for the refolded protein can dramatically improve the overall yield of protein from a refolding protocol. Interestingly, we found that the best pH and additives for refolding generally have differences with the buffer that is optimal to stabilize the refolded protein. For HA-RBD, sodium citrate was shown to stabilize the refolded protein after the arginine was removed and that concentration of HA-RBD to over 0.5 mg/mL was not possible in the absence of sodium citrate. However, citrate was not observed to have a significant effect on the refolding of HA-RBD, as judged from the DGR signals. MDM2 was more stable at pH 8, rather than in the refolding buffer at pH 8.5 and 1 mM of the divalent metal CaCl_2_ also helped. Again, CaCl_2_ was not identified to be a major contribution to the refolding of MDM2, as judged from the results of the additive screen. Additionally, the optimal pH for stabilizing IL-17A was found to be at pH 6, rather than refolding pH of 9.5. Based on our results, we concluded that while the refolding buffer can refold the protein into its native state, it does not necessarily mean that the refolding buffer is optimal for long term-protein stability. Therefore, screening for a protein stabilization buffer screen is critical after refolding the protein. It is possible that a buffer condition that supports the transition from an unfolded to a folded state (eg. by stabilizing an intermediate state) may act in a destabilizing manner on a folded for exactly the same reasons. Thus, we believe that many refolding protocols could be significantly improved by assaying for buffers that stabilize the final refolding state and that the combination of DGR and TSA provides a rapid mechanism to perform these analysis.

Our data further demonstrates the utility of DGR in establishing refolding conditions as pioneered by Biter *et al*. We have further expanded their approach to include an analysis of critical factors, such as time, redox couples, buffer composition and a secondary TSA to increase the chances of a successful refolding experiment.
